# Diabetoporosis: Role of nitric oxide

**DOI:** 10.17179/excli2021-3541

**Published:** 2021-04-16

**Authors:** Nasibeh Yousefzadeh, Sajad Jeddi, Khosrow Kashfi, Asghar Ghasemi

**Affiliations:** 1 Endocrine Physiology Research Center, Research Institute for Endocrine Sciences, Shahid Beheshti University of Medical Sciences, Tehran, Iran; 2 Department of Molecular, Cellular, and Biomedical Sciences, Sophie Davis School of Biomedical Education, City University of New York School of Medicine, NY, USA; 3 PhD Program in Biology, City University of New York Graduate Center, New York,NY, USA

**Keywords:** Diabetoporosis, nitric oxide, diabetoporosis

## Abstract

Diabetoporosis, diabetic-related decreased bone quality and quantity, is one of the leading causes of osteoporotic fractures in subjects with type 2 diabetes (T2D). This is associated with lower trabecular and cortical bone quality, lower bone turnover rates, lower rates of bone healing, and abnormal posttranslational modifications of collagen. Decreased nitric oxide (NO) bioavailability has been reported within the bones of T2D patients and can be considered as one of the primary mechanisms by which diabetoporosis is manifested. NO donors increase trabecular and cortical bone quality, increase the rate of bone formation, accelerate the bone healing process, delay osteoporosis, and decrease osteoporotic fractures in T2D patients, suggesting the potential therapeutic implication of NO-based interventions. NO is produced in the osteoblast and osteoclast cells by three isoforms of NO synthase (NOS) enzymes. In this review, the roles of NO in bone remodeling in the normal and diabetic states are discussed. Also, the favorable effects of low physiological levels of NO produced by endothelial NOS (eNOS) versus detrimental effects of high pathological levels of NO produced by inducible NOS (iNOS) in diabetoporosis are summarized. Available data indicates decreased bone NO bioavailability in T2D and decreased expression of eNOS, and increased expression and activity of iNOS. NO donors can be considered novel therapeutic agents in diabetoporosis.

## Introduction

The prevalence of type 2 diabetes (T2D) is increasing worldwide and is estimated to reach 693 million by 2045 (Guariguata et al., 2014[50]; Cho et al., 2018[29]). Diabetoporosis that is diabetic-related changes in bone, characterized by decreased bone quality and quantity (Ferrari et al., 2018[41]), is one of the leading causes of osteoporotic fractures in subjects with T2D (Wongdee and Charoenphandhu, 2011[164]). A higher risk of osteoporotic fractures in T2D patients has been reported in several population-based studies (Forsén et al., 1999[42]; de Liefde et al., 2005[36]; Ahmed et al., 2006[3]; Lipscombe et al., 2007[94]). A meta-analysis of case-control and cohort studies from 1980 to 2016 indicates that the risk of osteoporotic fractures is 50-80 % higher in an individual with T2D (Janghorbani et al., 2007[72]; Vestergaard, 2007[151]; Moayeri et al., 2017[101]). In addition, it has been shown that osteoporotic fractures increase the 1-year mortality rate by 15-20 % in the elderly (Johnell and Kanis, 2004[75]; Wang et al., 2013[152]; González‐Zabaleta et al., 2016[49]). These data emphasize the need for developing new prevention/treatment strategies against osteoporotic fractures in patients with T2D.

Accumulating evidence indicates that decreased nitric oxide (NO) bioavailability can contribute to diabetoporosis. NO is produced in the osteoblast and osteoclast cells by the three isoforms of NO synthase (NOS) enzymes (Ralston et al., 1994[130]; Armour and Ralston, 1998[10]; Klein-Nulend et al., 1998[89]; Mancini et al., 2000[97]). In T2D, within the bone cells, the expression and activity of the endothelial NOS (eNOS) are decreased (Kalyanaraman et al., 2018[80]), while that of the inducible isoform, iNOS, is increased (MacPherson et al., 1999;[96] Bhatta et al., 2016[15]). eNOS-derived NO increases osteoblastic bone formation (Tai et al., 2007[142]; Jamal and Hamilton, 2012[71]) and directly inhibits osteoclast-mediated bone resorption (Wimalawansa, 2000[159][161]). In contrast, iNOS-derived NO inhibits osteoblast-mediated bone formation and a stimulatory effect on osteoclast-mediated bone resorption (Damoulis and Hauschka, 1997[35]; Hof and Ralston, 2001[61]; van't Hof et al., 2004[150]). It has been reported that eNOS deficiency decreases the rate of bone formation (Aguirre et al., 2001[2]; Armour et al., 2001[10]; Wimalawansa, 2009[156]), accelerates osteoporosis (Wimalawansa, 2010[158]), delays the bone healing process, and increases the risk of bone fractures (Hof and Ralston, 2001[61]; Jamal and Hamilton, 2012[71]). Also, it has been reported that NO donors have protective effects against osteoporotic bone fractures in postmenopausal women (Jamal et al., 2004[70]; Rejnmark et al., 2006[131]; Pouwels et al., 2010[122]) and in ovariectomized and corticosteroid-treated rats (Wimalawansa et al., 1996[163]; Samuels et al., 2001[136]). The role of NO on the function of the bone in the normal state has been previously reviewed (Kalyanaraman et al., 2016[78], 2018[80]). Here, we review the role of NO in diabetoporosis.

## NO in the Bone

NO is produced in the cells of the bone by all three NOS isoforms, that is, eNOS, neural NOS (nNOS), and iNOS (Saura et al., 2010[138]). eNOS and nNOS are constitutively expressed and thus continuously produce low levels of NO. iNOS, on the other hand, is activated by certain stimuli, including proinflammatory cytokines, and produces high and biologically toxic concentrations of NO (Saura et al., 2010[138]). Effects of NO on bone function depend on its concentration (Joshua et al., 2014[76]), low physiological levels of NO have a stimulatory effect on normal bone formation (Ralston et al., 1995[129]; Wimalawansa, 2007[160]), development (Zaragoza et al., 2006[168]; Saura et al., 2010[138]), remodeling (Wimalawansa et al., 2000[157]), and fracture healing. In contrast, a pathologically high level of NO has inhibitory effects on all of these processes. 

### Expression and activity of eNOS in bones 

The eNOS gene is constitutively expressed in osteoblasts and osteocytes in both the fetus and adults (Helfrich et al., 1997[57]; Fox and Chow, 1998[44]). Furthermore, eNOS is also expressed in osteoclasts, bone marrow stromal cells, and chondrocytes of the epiphyseal growth plate (Mancini et al., 2000[97]; Wimalawansa, 2009[156]). Studies in rodents with targeted deletion of the eNOS gene have shown that eNOS-derived NO mediates the stimulatory effects of sex-steroid (Armour and Ralston, 1998[10]; Wimalawansa, 2010[158]), thyroid hormones (Kalyanaraman et al., 2014[81]), and mechanical loading on bone formation (Punjabi et al., 1992[125]; Fox et al., 1996[43]; Fox and Chow, 1998[44]). eNOS-deficient rodents show reduced prenatal and postnatal trabecular bone volume and cortical thickness, bone length, and bone mineral density (Armour et al., 2001[10]; Hefler et al., 2001[55]). In addition, eNOS-deficient mice have lower osteoblast (Afzal et al., 2004[1]) and higher osteoclast activities (Kasten et al., 1994[86]; Armour et al., 1999[10]; Percival et al., 1999[116]), thus presenting with a higher risk of osteoporotic fracture (Yan et al., 2010[165]) and lower rates of the bone healing process (Collin-Osdoby et al., 1995[30]) (Table 1(Tab. 1)).

### Expression and activity of iNOS in bones 

In neonatal female rats, the iNOS gene was shown to be constitutively expressed in osteoblasts (Hukkanen et al., 1999[65]). However, under normal conditions, iNOS is not detectable in adults; pro-inflammatory cytokines induce its expression and activity in osteoclasts and pre-osteoclast cells (Brandi et al., 1995[19]; Zheng et al., 2006[171]; Wimalawansa, 2008[162]). iNOS-deficient mice do not have any apparent bone abnormalities during their adult life, but they have lower prenatal bone growth and bone length (Watanuki et al., 2002[154]) (Table 1(Tab. 1)). High concentrations of NO that is produced by iNOS inhibit the activity and proliferation of osteoblasts (Damoulis and Hauschka, 1997[35]; Hof and Ralston, 2001[61]; van't Hof et al., 2004[150]) and increases osteoclast activity in pathophysiological conditions (Mundy, 1993[105]; Chen et al., 2002[27], 2005[26]; Hao et al., 2005[54]; Ho et al., 2005[60]; Wimalawansa, 2008[162]; Rajfer et al., 2019[128]).

### Expression and activity of nNOS in bones 

Some studies have failed to detect nNOS expression in the bone cells (Schmidt et al., 1992[139]; Helfrich et al., 1997[57]); however, nNOS expression has been reported in bone lining cells and in osteocytes (Helfrich et al., 1997[57]; Fox and Chow, 1998[44]) during skeletal development (Hukkanen et al., 1999[65]) and fracture healing (Zhu et al., 2001[172]). nNOS-deficient mice have higher trabecular and cortical bone mineral density and lower bone remodeling with lower numbers of osteoclasts and osteoblasts (Jung et al., 2003[77]; van't Hof et al., 2004[150]) (Table 1(Tab. 1)).

## Diabetoporosis at a Glance

Despite having a normal or increased bone mineral density, T2D patients are at a higher risk of osteoporotic fractures (van Daele et al., 1995[149]; Sosa et al., 1996[140]; Bonds et al., 2006[16]). This paradox suggests that the etiology of osteoporotic fractures in T2D is different from that of the general population (Jindal et al., 2018[73]). According to a meta-analysis of association studies, the higher risk of osteoporotic fractures in T2D patients is associated with lower trabecular bone quality, that is incomplete, poorly connected, and widely spaced trabeculae (Ho-Pham and Nguyen, 2019[63]), and also with lower cortical bone quality, encompassing lower width and higher porosity (Patsch et al., 2013[115]). In addition, a lower bone turnover rate (Hygum et al., 2017[66]; Purnamasari et al., 2017[126]; Napoli et al., 2018[108]), a higher degree of mineralization (Pritchard et al., 2013[123]), a lower rate of bone healing (Norris and Parker, 2011[112]), and abnormal posttranslational modifications of collagen (Picke et al., 2019[120]) have been reported in diabetoporosis.

**Figure 1:**
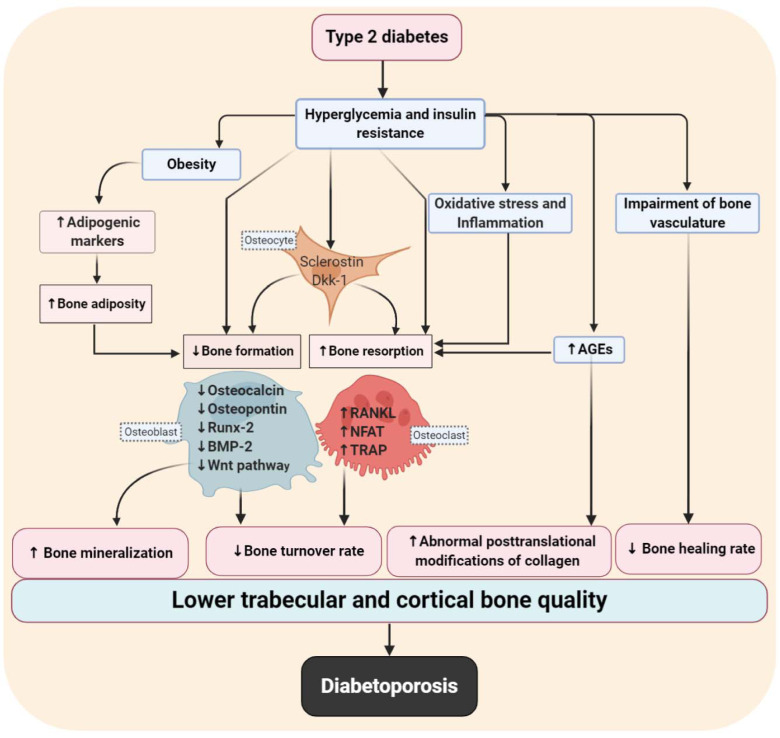
Main pathophysiological mechanisms involved in diabetoporosis. Type 2 diabetes (T2D) decreases trabecular and cortical bone quality by decreasing bone turnover and healing rates and increasing bone mineralization and abnormal posttranslational modifications of collagen.

The pathophysiological mechanisms of diabetoporosis are quite complex but can be divided into direct and indirect effects (Palermo et al., 2017[114]). In addition to the direct effects of hyperglycemia and insulin resistance on bone quality (Figure 1(Fig. 1)), the increased risk of osteoporotic fractures may also be explained by the presence of diabetic complications, decreased physical activity, obesity, lower vitamin D levels, and a higher risk of falls (Oei et al., 2015[113]). Bone vasculature impairment, increased inflammation, oxidative stress (McFarlane et al., 2004[100]; Hofbauer et al., 2007[62]; Kalyanaraman et al., 2018[80]), and bone marrow adiposity (Costantini and Conte, 2019[32]) are key factors that contribute to higher incidences of osteoporotic fractures and delayed fracture healing in T2D (Figure 1(Fig. 1)).

As shown in Figure 1(Fig. 1), hyperglycemia and insulin resistance directly decrease osteoblast differentiation and activity by decreasing the expression of osteoblast-related markers, including the Runt-related transcription factor 2 (Runx-2), osteocalcin, bone morphogenetic protein-2 (BMP-2), osteopontin. Wnt signaling pathway is also suppressed (Inaba et al., 1995[68]; Chiu et al., 2004[28]; Mathieu et al., 2005[98]; Hamann et al., 2011[53]; Sarkar and Choudhury, 2013[137]; Lattanzio et al., 2014[90]; Perez-Diaz et al., 2015[117]; Wei et al., 2015[155]), osteoclast activation and differentiation are increased through increases in the expression of osteoclast-related markers including the nuclear factor of activated T cells (NFAT), receptor activator of nuclear factor-kappa-Β ligand (RANKL), and tartrate-resistant acid phosphatase (TRAP) (McFarlane et al., 2004[100]; Hofbauer et al., 2007[62]; Picke et al., 2016[121]; Kalyanaraman et al., 2018[80]). In addition, increasing serum concentrations of the Wnt inhibitors, sclerostin, and Dickkopf WNT signaling pathway inhibitor-1 (DKK-1) by osteocytes can decrease bone turnover rate in T2D. Hyperglycemia and insulin resistance also indirectly increase the expression of adipogenic markers such as peroxisome proliferator-activated receptor γ (PPAR-γ) in bones and have inhibitory effects on the activity and differentiation of osteoblasts by increasing fat accumulation in the marrow cavity of long bones. In addition, hyperglycemia and insulin resistance indirectly affect bone quality by increasing advanced glycation end products (AGEs), oxidative stress, inflammation, and impaired bone vasculature. These changes might explain the higher risk of bone fractures and osteoporosis and the lower rate of bone healing in T2D.

## NO Bioavailability in Diabetic Bones

**Figure 2:**
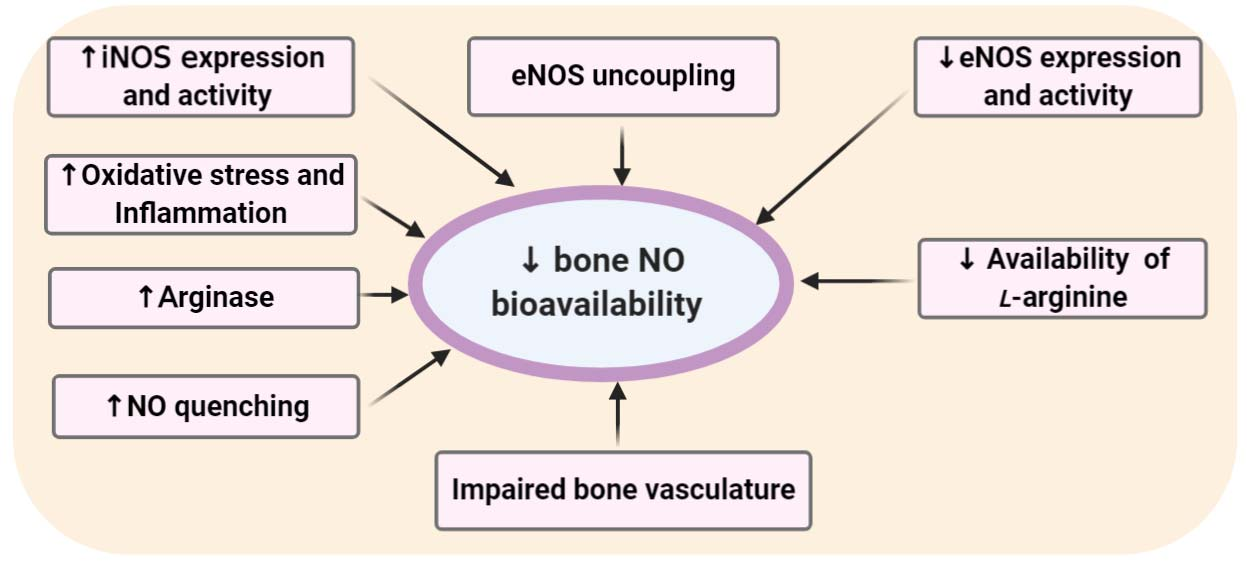
Proposed mechanisms involved in decreased endothelial nitric oxide (eNOS)-derived NO bioavailability and activity in bones of type 2 diabetic subjects

Decreased NO bioavailability has been reported in bones of humans and animals with T2D and can be considered as one of the main mechanisms in diabetoporosis. As shown in Figure 2(Fig. 2), lower eNOS expression (Kalyanaraman et al., 2018[79]) or activity (Mordwinkin et al., 2012[103]) resulting in diminished NO synthesis and increased NO oxidation due to NO quenching by AGEs (Bucala et al., 1991[20]; Alikhani et al., 2007[5]) are the leading causes of decreased NO bioavailability in the diabetic bone. In addition, reduced availability of L-arginine, the substrate for the NOS enzymes, increases arginase activity (Bhatta et al., 2016[15]); increased expression and activity of iNOS (MacPherson et al., 1999[96]), impaired vasculature of the bones (Stabley et al., 2015[141]), uncoupling of eNOS (Kalyanaraman et al., 2018[80]), and damaged to the eNOS-caveolin-1 complex (Aicher et al., 2003[4]; Cao et al., 2012[22]) may be involved in decreased NO bioavailability in the diabetic bones. 

eNOS uncoupling in the bones of T2D patients is at least in part due to increased production of bone morphogenetic protein 4 (BMP4) (Youn et al., 2015[167]) that leads to an eNOS-mediated superoxide production (Thum et al., 2007[146]). Lower activity of the eNOS/cGMP/PKG pathway due to the uncoupling of eNOS, inhibition of guanylate cyclase activity, and suppression of PKG transcription have all been reported in diabetic bones (Kalyanaraman et al., 2018[80]). In T2D, endothelial progenitor cells synthesize less NO because of the damaged eNOS-caveolin-1 complex (Aicher et al., 2003[4]; Cao et al., 2012[22]) that is associated with increased serum levels of Dickkopf-1, which is an inhibitor of osteoblast differentiation (Lattanzio et al., 2014[90]).

### Bone remodeling in diabetes

Bone remodeling is a life-long process and is achieved within anatomical structures that are known as a basic multicellular unit (BMU). These provide a unique microenvironment to facilitate coupled bone resorption and formation (Andersen et al., 2009[7]; Raggatt and Partridge, 2010[127]). Bone remodeling has four consecutive steps including activation, resorption, reversal, and formation, which require a coordinated action of the bone cells, including osteocytes, osteoblasts, osteoclasts, and bone-lining cells (Feng and McDonald, 2011[40]).

Step 1 (activation), the osteocytes sense signals for initiating remodeling; these include mechanical forces, changes in calcium homeostasis, or changes in hormone levels that translate into biological signals (Bonewald, 2007[17]; Raggatt and Partridge, 2010[127]). In osteocytes, initiating bone remodeling signals inhibit the expression of transforming growth factor β (TGF-β, as an inhibitor of bone resorption) (Heino et al., 2002[56]; Raggatt and Partridge, 2010[127]), and with a delay of about 5 days inhibit the expression of sclerostin (SOST, an inhibitor of bone formation) (van Bezooijen et al., 2004[148]; Li et al., 2005[92]; Robling et al., 2008[132]; Gasser and Kneissel, 2017[46]). In addition, the bone lining cells create a raised canopy above the remodeling surface, which merges with the bone vasculature for recruitment of osteoclast and osteoblast progenitor cells to the BMU (Arias et al., 2018[8]). 

Step 2 (resorption), the decreased TGF-β in osteocytes recruits hematopoietic stem cells (HSC) from the bone marrow or the circulation; these HCSs are then differentiated to osteoclasts in the presence of monocyte/macrophage colony-stimulating factor (M-CSF) and the RANKL (Boyle et al., 2003[18]). Low levels of TGF-β increase the RANKL/osteoprotegerin (OPG) ratio and M-CSF expression in preosteoblasts (Karst et al., 2004[85]). OPG negatively regulates RANKL binding to RANK that is essential for activation and differentiation of osteoclasts (Karst et al., 2004[85]). In this step, osteoclasts digest organic and inorganic bone matrices by secreting acid phosphatase, cathepsin K, and collagenase, a process known as bone resorption (Henriksen et al., 2011[58]). 

Mononuclear macrophage-like cells in step 3 (reversal) engulf and remove demineralized undigested collagen and generate transition signals that stop bone resorption and start bone formation (Raggatt and Partridge, 2010[127]). 

**Figure 3:**
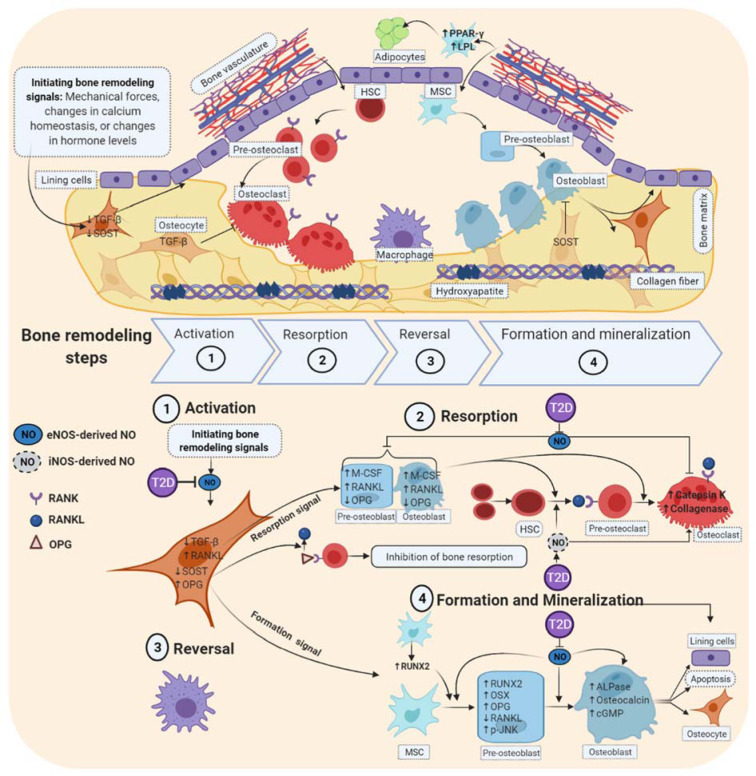
Effects of type 2 diabetes on bone remodeling: role of nitric oxide. T2D decreases osteoblastic bone formation and has a stimulatory effect on osteoclast-mediated bone resorption. These effects are mediated in part by a decrease in eNOS-derived NO and an increase in iNOS-derived NO.

Step 4 (formation and mineralization), in response to a decrease in SOST within osteocytes, mesenchymal stem cells (MSC) are recruited and differentiated into osteoblasts that start the bone formation and mineralization process. When an equal quantity of resorbed bone has been replaced, the remodeling cycle is terminated (Franz‐Odendaal et al., 2006[45]). Some osteoblasts in this step undergo apoptosis, others turn into lining cells, still, others remain trapped within the bone matrix and become osteocytes (Figure 3(Fig. 3)). 

In T2D and the bone remodeling process, there is a decrease in eNOS-derived NO and an increase in iNOS-derived NO; this leads to inhibition of steps 1 and 4, activation and bone formation, respectively; and at the same time, step 2, bone formation is stimulated. As shown in Figure 3(Fig. 3), T2D decreases the production of eNOS-derived NO in osteocytes and, therefore, decreases osteocytes' capabilities in detecting and initiating the bone remodeling signals (step 1) (Collin-Osdoby et al., 2000[31]; Bakker et al., 2009[14]). eNOS-derived NO increases in response to mechanical forces, thyroid hormones, and estrogens (Fox et al., 1996[43]; Armour and Ralston, 1998[10]; Kalyanaraman et al., 2014[81]). T2D by decreasing eNOS-derived NO and increasing iNOS-derived NO increases bone resorption (step 2). eNOS-derived NO in T2D inhibits the production of M-CSF and RANKL and stimulates the production of OPG in both preosteoblasts and osteoblasts; these effects result in a decrease in recruitment of HSC and their differentiation to osteoclast (Wongdee and Charoenphandhu, 2011[164]; Catalfamo et al., 2013[23]). In addition, eNOS-derived NO decreases the activities of cathepsin K, a marker of high bone resorption and collagenase in osteoclast (Percival et al., 1999[116]; Gyurko et al., 2005[51]; Alselami et al., 2015[6]). Therefore, T2D increases bone resorption by decreasing eNOS-derived NO (Pezhman et al., 2019[119]). T2D stimulates the production of iNOS-derived NO, which increases PPARγ production by HSC and, therefore, stimulates differentiation of HSC to osteoclasts; in addition, iNOS-derived NO increases the activities of cathepsin K and collagenase and osteoclast activity (Percival et al., 1999[116]; Gyurko et al., 2005[51]; Alselami et al., 2015[6]). These effects result in increased bone resorption. 

eNOS-derived NO directly activates and facilitates osteoblastic differentiation from MSC (Hikiji et al., 1997[59]) through phosphorylation of JNK/MAPK in preosteoblasts (Yang et al., 2018[166]). After transportation to the nucleus, p-JNK induces the expression of osteogenic transcription factors such as Runx2, osterix (OSX), and osteopontin (OPN) (Aguirre et al., 2001[2]) and represses the expression of adipogenic transcription factors such as PPARγ and lipoprotein lipase (LPL), thus increasing osteogenesis and decreasing adipogenesis (Rosen et al., 1999[133]; Aguirre et al., 2001[2]; Zhao et al., 2016[170]; Yang et al., 2018[166]). In addition, eNOS-derived NO directly activates osteoblast activity by increasing the alkaline phosphatase (ALPase) (Inoue et al., 1995[69]) and osteocalcin levels (Pun et al., 1989[124]) as well as increasing intracellular concentrations of cGMP (Hagiwara et al., 1996[52]). 

## Treatment of Diabetoporosis by Nitric Oxide

Available treatments for osteoporosis are limited by cost, side effects, and efficacy, with limited impact on the cortical bone (Table 2(Tab. 2); References in Table 2: Austin and Heath, 1981[12]; Burr et al., 2015[21]; Chau and Edelman, 2002[24]; Chen et al., 2014[25]; Dai et al., 2020[33]; Fabre et al., 2020[38]; Gattereau et al., 1980[47]; Ilich and Kerstetter, 2000[67]; John et al., 2011[74]; Kanazawa, 2017[82]; Karras et al., 2018[84]; Khosla and Hofbauer, 2017[88]; Khosla et al., 2007[87]; Li et al., 2018[91]; Lim and Bolster, 2017[93]; Mauvais-Jarvis et al., 2013[99]; Mohsin et al., 2019[102]; Morita et al., 2016[104]; Neer et al., 2001[109]; Nemeth and Goodman, 2016[111]; Nemeth et al., 2001[110]; Pettway et al., 2008[118]; Russell, 2011[134]; Russo and Russo, 2006[135]; Takakura et al., 2017[143]; Tella and Gallagher, 2014[144]; Tella et al., 2017[145]; Tsourdi et al., 2017[147]; Ward and Rauch, 2018[153]; Zhang et al., 2020[169]). Therefore, there is a need for easily administered and inexpensive agents that increase bone trabecular and cortical strength and decrease the risk of osteoporotic fractures. NO donors have a high potential to be cost‐effective novel therapeutic agents against osteoporosis and, in particular, against diabetoporosis.

### Possible strategies for the treatment of diabetoporosis by nitric oxide

Organic nitrates are used for treating heart failure and hypertension; epidemiological studies have shown that their use can reduce the risk of osteoporotic fractures (Rejnmark et al., 2006[131]; Pouwels et al., 2010[122]). Based on these observations, the protective effects of organic nitrates against osteoporotic fractures were reported in ovariectomized and corticosteroid-treated rats (Wimalawansa et al., 1996[163]; Samuels et al., 2001[136]; Wimalawansa, 2007[160], 2009[156]), mice (Wimalawansa et al., 1996[163]; Hukkanen et al., 2003[64]), and in ovariectomized (Wimalawansa, 2000[159]; Nabhan and Rabie, 2008[107]) and postmenopausal women (Wimalawansa et al., 1996[163]). Organic nitrates stimulate osteoblast-mediated bone formation (Wimalawansa et al., 1996[163]; Wimalawansa, 2000[161]) and inhibit osteoclast-mediated bone resorption (Fan et al., 2004[39]), thus decreasing the risk of osteoporotic fractures. Organic nitrates are the only FDA-approved NO donors, but their potential benefits are rapidly lost on long-term use due to the possible development of tolerance and endothelial dysfunction (Daiber and Münzel, 2015[34]). Inorganic nitrites and nitrates are NO donors with strong NO-like effects in both animals and humans; it has been suggested that they can act as suitable alternatives to organic nitrates (Münzel and Daiber, 2018[106]). These agents can protect against diabetoporosis directly by decreasing osteoclast activity and increasing osteoblast activity (see section “NO in the bone”), or indirectly, by improving the metabolic status (Ghasemi and Jeddi, 2017[48]; Lundberg et al., 2018[95]; Kapil et al., 2020[83]) and decreasing body weight (Bahadoran et al., 2020[13]).

## Conclusion and Future Perspective

Decreased bone NO bioavailability in T2D is one of the primary mechanisms underlying diabetoporosis. This reduced NO bioavailability is due to decreased expression of eNOS, availability of L-arginine, and activity of cGMP/PKG, as well as increased eNOS uncoupling, expression, and activity of iNOS and arginase. NO donors can potentially be used as safe and cost‐effective novel therapeutic agents in diabetoporosis. This issue, however, remains to be verified in a well-designed clinical trial. 

## Notes

Khosrow Kashfi and Asghar Ghasemi (Endocrine Physiology Research Center, Research Institute for Endocrine Sciences, Shahid Beheshti University of Medical Sciences, No. 24, Parvaneh Street, Velenjak, P.O. Box: 19395-4763, Tehran, Iran; E-mail: Ghasemi@endocrine.ac.ir) contributed equally as corresponding author.

## Acknowledgements

This study was supported by Shahid Beheshti University of Medical Sciences [grant No. 27443-1], Tehran, Iran.

## Conflict of interest

The authors declare that they have no competing interests.

## Figures and Tables

**Table 1:**
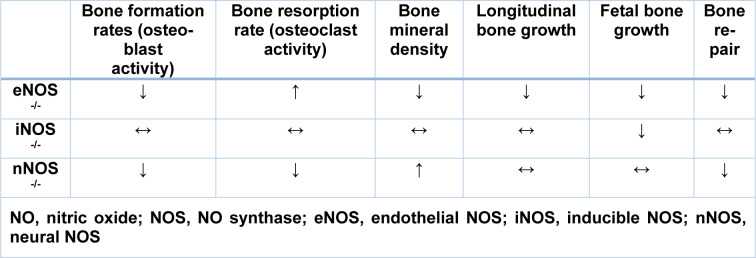
The bone phenotypes in NOS-deficient rats and mice (Diwan et al., 2000; Armour et al., 2001; Hefler et al., 2001; Watanuki et al., 2002; Jung et al., 2003; van't Hof et al., 2004; Yan et al., 2010)

**Table 2:**
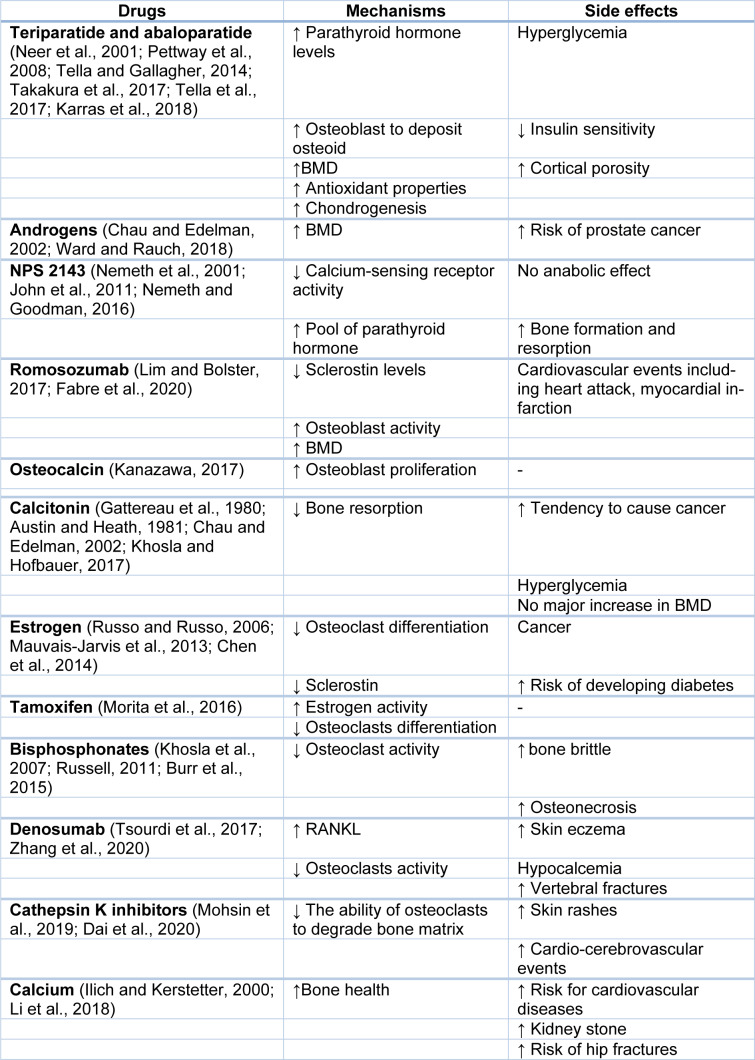
Mechanisms and side effects of current drugs used in treating of osteoporosis
